# Impact of Ivabradine on renal function in septic patient with early renal impairment

**DOI:** 10.1186/s42077-021-00162-6

**Published:** 2021-07-13

**Authors:** Amr Sobhy, Lobna A. Saleh, Aktham Adel Shoukry

**Affiliations:** 1 Department of Anaesthesiology, Intensive Care and Pain Management, Faculty of Medicine, Ain-Shams University, Cairo, 11566 Egypt; 2 Department of Clinical Pharmacology, Faculty of Medicine, Ain-Shams University, Cairo, Egypt

**Keywords:** Mortality, Ivabradine, Sepsis, Cystatin c, Renal impairment

## Abstract

**Background:**

Acute kidney injury (AKI) with sepsis increases mortality significantly. The pathophysiology of AKI during sepsis is complex and multifactorial. Lower heart rate is associated with better survival in patients with multiple organ dysfunction syndrome (MODS), a disease mostly caused by sepsis. In our study, we hypnotized that use of ivardrabine as heart rate reducing agent in septic patient with renal impairment may improve renal function.

**Results:**

Fifty patients with sepsis with early renal impairment were divided in 1: 1 ratio to receive Ivabradine (group I) or not (group C). The average age of the included patients was almost 45 years, chest disorders were the main cause of sepsis in both groups. There were statistically significant differences between both groups in terms of reduction of heart rate group (I) (68.13 ± 3.34) versus (group C) (87.04 ± 3.23) and (*P* < 0.001) also, improvement in eGFR by Cystatin c in group (I) (103.32 ± 6.96) versus (group C) (96.25 ± 6.36) and (*P* < 0.001) also vasopressor dosage consumption (*P* < 0.001). As regards secondary outcomes, there were no statistically significant differences between study’s groups in terms of length of hospital stay (*P* = 0.390), need for hemodialysis (*P* = 0.384), and mortality (*P* = 1.000).

**Conclusions:**

We concluded that Ivabradine as an adjuvant therapy in septic patients with renal impairment is promising agent to reduce such impairment.

**Trial registration:**

Pan African Clinical Trial Registry: Identification number for the registry is PACTR201911806644230.

**Supplementary Information:**

The online version contains supplementary material available at 10.1186/s42077-021-00162-6.

## Background

The incidence of sepsis and renal impairment in critical patients is gradually increasing, and both of them indicate a poor prognosis and increases mortality significantly (Schrier and Wang 2004).

In critically ill patients, serum cystatin C seems to be an early and efficient marker for renal dysfunction. Especially with mild reductions in GFR, it is a better predictor for the development of renal failure than plasma creatinine (Royakkers et al. 2007).

The pathophysiology of AKI during sepsis is complex and multifactorial and involves changes of renal hemodynamics, endothelial dysfunction, renal parenchymal inflammatory cell infiltration, intraglomerular thrombosis, and congestion of tubules by waste and necrotic cells (Wan et al. 2008).

Lower heart rate is associated with better survival in patients with multiple organ dysfunction syndrome (MODS), a disease mostly caused by sepsis (de Castilho et al. 2017). Controlling sinus tachycardia with ivabradine (a specific inhibitor of the If current in the Sino atrial node) was effective in reducing microvascular derangements evoked by experimental sepsis, which was accompanied by less organ dysfunction. These results suggest that ivabradine yields beneficial effects on the microcirculation of septic animals (Miranda et al. 2017).

In our study, we hypnotized that use of ivardrabine as heart rate reducing agent in septic patient with renal impairment may improve renal function.

## Methods

After informed consent and after approval of institutional ethical com, this prospective randomized study conducted on 50 patients ranging age 18–60 years old diagnosed with severe sepsis ≤ 24 h according to Surviving Sepsis Campaign Guidelines Committee (Rhodes et al. 2017) with sinus rhythm with heart rate ≥ 95 bpm (after adequate resuscitation) and early renal impairment defined by the RIFLE classification (Bellomo et al. 2004) (when increased plasma creatinine × 1.5 or GFR decrease > 25% of normal range for age or urine output < 0.5 mL/kg/h × 6 h).

### Exclusion criteria

Patients with pre-existing renal or started renal dialysis, hypersensitivity to the drug, pregnancy, severe hepatic (class C according to Child Pugh Classification), or cardiac insufficiency (class 6 according to New York Heart Association (NYHA) Functional Classification

Sick sinus syndrome, sinu-atrial block pacemaker-dependency, 3rd degree AV block, use antifungals of the azole-type (ketoconazole, itraconazole), macrolide antibiotics (clarithromycin).

Patient with positive swab for COVID 19 (pathophysiology still unclear).

Our primary outcome is to determine whether the reduction of heart rate below the predefined threshold of 95/min by using ivabradine could improve the kidney function by 25% within 72 h from the start of treatment.

Secondary outcome is to assess prevention of dialysis, vasopressor dosage consumption, ICU hospital stay, and mortality.

All patients diagnosed with early sepsis were be resuscitated according to our hospital protocol and after 24 h of hemodynamic optimization in order to establish an adequate circulating blood volume (adjusted by central venous pressures of ≥ 13 mmHg, a mixed venous oxygen saturation higher than 65% (SvO_2_), and mean arterial pressure MAP of 65 mmHg with or without vasopressor support or higher, serum lactate ≤ 6 mmol/l)

Patients having persisted heart rate 95/min or higher and early renal impairment (when increased plasma creatinine × 1.5 or GFR decrease > 25% of normal range for age or urine output < 0.5 mL/kg/h × 6 h) enrolled in our study and randomly divided using a closed sealed envelope method of randomization into two groups

Group C: received conventional treatment sepsis according to ICU protocol and placebo tablet.

Group I: received conventional treatment sepsis according to ICU protocol and enteral preparation (orally, via nasogastric tube) of Ivabradine Procoralan® 5 mg (Manufacturer, Servie (Ireland) Industries Ltd Gorey Roa Arklow—Co. Wicklow Ireland) for 3 days The dosing is started at 5 mg twice daily, and if tolerated can be safely continued to 7.5 mg twice daily if heart rate don’t decrease after third dose.

All patients were attached to standard monitoring upon arrival to ICU. Ultrasound-guided central venous catheter and arterial catheter was inserted. The correct positioning of the venous catheter tip was confirmed by chest X-ray examinations (between superior vena cava and right atrium).

Baseline laboratory were taken, complete blood picture, C.R.P, liver function

Renal assessment was performed utilizing the following:

- Urine protein-to-creatinine ratio, BUN, serum creatinine, serum cystatin C by ELISA, and GFR were estimated for each patient using the following equations: (Grubb 2000)

Simplified modification of diet in renal disease equation:

$$ \mathrm{eGFR}\ \left(\mathrm{mL}/\min /1.37\mathrm{m}2\right)=186.3\times \left[\mathrm{serum}\ \mathrm{creatinine}\ \mathrm{mg}/\mathrm{dl}\right)\Big]-1.154\ \left[\mathrm{age}\left(\mathrm{years}\right)\right]-0.203\times (0.742). $$

Equations estimating GFR based on cystatin C:

$$ \mathrm{GFR}=76.6\times \mathrm{Cystatin}\ \mathrm{C}\ {\left(\mathrm{mg}/\mathrm{l}\right)}^{-1.16}. $$

All patients were resuscitated according to our hospital protocol; initial resuscitation a minimum of 30 mL/kg of crystalloids to achieve the following goal: MAP ≥ 65 mm Hg, urine output ≥ 0.5 mL/kg/h, CVP 13–12 mmHg and ScvO_2_ 70%. Cultures including blood cultures withdrawn before antimicrobial therapy if no delay in the start of antimicrobial. IV broad spectrum started.

If adequate fluid resuscitation therapy was not able to restore hemodynamic, vasopressor therapy was initiated to target a mean arterial pressure (MAP) of 65 mmHg. Norepinephrine as the first-choice vasopressor ((LEVOPHED®, norepinephrine bitartrate injection, USP, contains the equivalent of 6 mg base of LEVOPHED per each 6 mL ampule (1 mg/mL) CIBA Pharmaceuticals Company, USA). Were diluted by 50 mL D5% (1 mL equal 80 μg) 0.5–1 μg/kg/min.

If adequate fluid resuscitation and vasopressor therapy are not able to restore hemodynamic stability, IV hydrocortisone at a dose of 200 mg/day was given.

As regard renal impairment management optimization of volume status and avoidance of nephrotoxic medications for 72 h (Moore et al. 2018).

- Strict fluid balance to maintain urine output more than 0.5 mL/kg
- Avoid nephrotoxic drugs and adjusted dose of used drugs according to eGFR
- The Start a trial of intravenous furosemide, which could help manage his fluid overload

(d) Hemodialysis if

- Failed conservative management for 3 days (increased plasma creatinine × 3 or acute plasma creatinine ≥ 350 μmol/L or acute rise ≥ 44 μmol/L or urine output < 0.3 mL/kg/h × 24 h or anuria × 12 h
- Severe fluid overload
- Refractory hypertension
- Uncontrollable hyperkalemia
- Nausea, vomiting, poor appetite, gastritis with hemorrhage
- Lethargy, malaise, somnolence, stupor, coma, delirium, asterixis, tremor, seizures
- Severe metabolic acidosis
- Blood urea nitrogen (BUN) > 70–100 mg/dL
  Our end point would be if either of the following occurred:
- Failure to control the heart rate (sinus tachycardia) within 72 h
- Deterioration of kidney function within the 72 h of start of treatment with need of hemodialysis
- Occurrence of side effects related to the use of Ivabradine, e.g., (bradycardia, photophobia)

Measurements for patients of both groups: over 72 h

1. Heart rate
2. Daily urine output
3. Daily renal function (BUN-e GFR by Cystatin c and creatinine)
4. Consumption dose of norepinephrine
5. Daily serum lactate
  Most common side effects related to the use of Ivabradine will be monitored and managed accordingly, e.g., sever bradycardia or heart block the drug will be stopped and cardiology consultation sought. Emergency management with atropine and temporary pacemaker was applied if needed.

- Photophobia and blurring of vision usually transient and disappear after discontinue the drug.

Patients of both groups will be followed up

- Number of patients need hemodialysis
- I.C.U length of stay and mortality rate at 28 days

as regard sample size calculation:

It was calculated using PASS 11 program with the following parameter: sample size of 25 patients in each group achieving 87% power to detect a difference of (40%) in renal impairment rate after intervention assuming that rate in control group is (60%) and in intervention group (20%) with 0.05 significance level.

### Statistical analysis

Statistical analysis was performed using computer software statistical package for the social science (SPSS, version 22.0; SPSS Inc., Chicago, Illinois, USA)Description of quantitative (numerical) variables was performed in the form of mean ± SD. Description of qualitative (categorical) data was performed in the form of number of cases and percent. Error bars represent 95% confidence interval.

Analysis of unpaired numerical variable was performed using the unpaired Student *t*-test, whereas analysis of paired numerical variables was performed using repeated measure general linear model analysis of variance.

Analysis of categorical data was performed using Fisher’s exact test or the χ2-test, whenever appropriate.

The significance level was set at *P* value of 0.05 or less, and *P* value of 0.01 or less was considered highly significant.

## Results

**Fig. 1:**
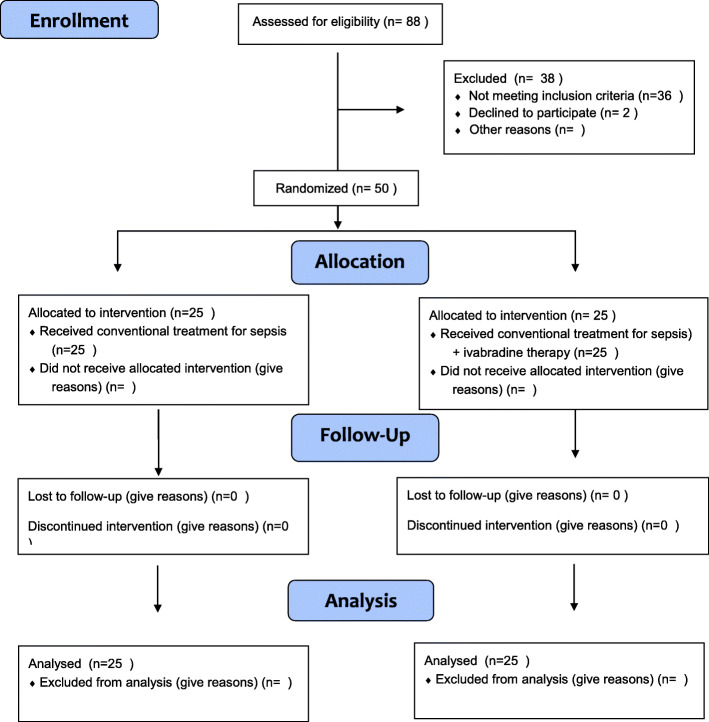
Enrollment

This randomized, double-blinded prospective study was conducted from December 2019 to October 2020; after hemodynamic optimization, we screened 88 patients with 38 being excluded due to heart rate values of less than 95 min (*n* = 16) or on renal dialysis (*n* = 10) or COIVD positive (*n* = 10). In another 2 patients, we could not obtain informed consent. Thus, a total of 50 patients were included and randomly assigned to the 2 study groups in a 1:1 ratio (Fig. 1).

The average age of the included patients was almost 45 years, chest disorders were the main cause of sepsis in both groups. Considering the baseline clinical parameters, there were no statistically significant differences between both groups in terms of SOFA score (*P* = 0.496), weight and causes of sepsis also no statistically significant differences between both groups after adequate resuscitation in terms of lactate level (*P* = 0.130), baseline heart rate (*P* = 0.369), (SvO_2_) (*P* = 0.414), MAB (mean 68) (*P* = 0.135), CVP (*P* = 0.139). As regard degree of renal impairment, there were no statistically significant differences between both groups in terms of baseline serum cystatin C (*P* = 0.123), estimated glomerular filtration rate based on cystatin C (mL/min/1.73 m^2^) (mean 44 (mL/min/1.73 m^2^) (*P* = 0.094), daily urine output (*P* = 0.840) (Table 1).

**Table 1:** Baseline data

	Group C (n=25)	Group I (n=25)	Tests	
			t/X^2^	P-value
**Age (years)**	44.16±10.54	45.32±9.78	0.404	0.688
**Weight (kg)**	83.60±8.23	85.32±9.42	0.688	0.495
**Cause of Sepsis**				
Pneumonia	12(48%)	13(52%)	0.267	0.966
Abdominal	8(32%)	7(28%)		
Necrotizing fasciitis	2(8%)	3(12%)		
Septic wound	2(12%)	2(8%)		
**Amount of fluid prior to inclusion**	5410.48±490.81	5394.00±398.98	0.130	0.897
**SOFA score (Median (IQR)**	9 (7 -12.25)	8(6.75-12)		0.496
**Optimization of adequate resuscitation**				
Serum lactate	1.96±0.46	2.15±0.44	1.542	0.130
MAB	68.20±1.29	67.56±1.66	1.522	0.135
CVP	9.72±1.28	10.24±1.16	1.505	0.139
(SvO2)	69.40±3.32	70.16±3.20	0.825	0.414
HERAT RATE	98.20±7.21	96.84±3.33	0.856	0.396
Serum Cystatin C (mg/l)	1.71±0.16	1.79±0.17	1.571	0.123
**eGFR** C (ml/min/1.73 m2)	44.28±2.88	45.76±3.24	1.706	0.094
**DAILY URINE OUTPUT**	844.52±107.76	838.00±118.52	0.204	0.840
**DAILY NOREPIEPHRINE :** 0.3-0.5 μg/Kg/Min	0.44±0.16	0.47±0.20	0.539	0.592

Group (C): received conventional treatment sepsis and placebo tablet, group (I): received conventional treatment sepsis and Ivardrabine tablet, (SvO2): mixed venous saturation, CVP: central venous pressure, MAP: mean arterial blood pressure, eGFR C :estimated glomerular filtration rate based on cystatin C

### The outcome results

Group (I) show significant reduction in heart rate started 24 h after administration of ivabradine therapy (87.04 ± 3.23) and marked reduction (68.13 ± 3.34) on day 3, and this reduction was highly statically significant (*P* < 0.001) in compare to baseline value (96.84 ± 3.33) in the same group also when compare both groups together.

There was significant reduction in dose in vasopressor in group (I) in day 2 (mean 0.27 ± 0.06) in comparison to group (C) (mean 0.33 ± 0.07).

*P* = 0.002*

As regards renal impairment, there was significant improvement in renal function in group (I) started from day 2 and continue in terms of daily urine output (mean 2141.96 ± 210.83) in day 3 in comparison to baseline value (829.60 ± 105.17) and when compared to group (C) (mean 1838.44 ± 131.88) also in terms of reduction in serum cystatin c level and increase in estimated GFR, and this reduction was highly statistically significant (*P* < 0.001).

Although the number of patients that need hemodialysis in group (I) was less than group (C) 6(13%), 13(16%) respectively but it was statically insignificant (*P* = 0.384).

Considering secondary outcomes, there were no statistically significant differences between study groups in terms of length of ICU stay and mortality rate (*P* = 0.390), (*P* = 1.000) respectively (Table 2).

**Table 2:** Outcome

	Group C (n=25)	Group I (n=25)	Tests	
	Mean±SD	Mean±SD	t/X^2^	P-value
**Heart rate (beat/min.)**				
T0	98.20±7.21	96.84±3.33	0.856	0.396
T1	93.32±5.78	87.04±3.23	4.742	<0.001**
T2	86.36±5.82	78.36±3.03	6.095	<0.001**
T3	84.64±5.03	68.8±3.34	13.111	<0.001**
**Urine output (ml/24 hrs.)**				
T0	834.44±88.50	829.60±105.17	0.176	0.861
T1	1102.48±165.75	1090.40±179.53	0.247	0.806
T2	1583.56±169.26	1684.60±169.40	2.110	0.040*
T3	1838.44±131.88	2141.96±210.83	6.103	<0.001**
**cystatin c (mg/l)**				
T0	1.71±0.16	1.79±0.17	1.571	0.123
T1	1.57±0.14	1.42±0.15	3.653	0.002*
T2	1.36±0.12	1.14±0.13	6.421	<0.001**
T3	1.21±0.15	0.98±.12	5.615	<0.001**
**eGFR (ml/min/1.73m**^**2**^**)**				
T0	44.68±3.67	44.24±3.38	0.441	0.661
T1	70.24±4.59	73.76±4.44	2.758	0.008*
T2	79.20±4.07	82.96±5.68	2.689	0.010*
T3	96.52±6.36	103.32±6.96	3.606	<0.001**
**Number need HD**	4(16%)	2(8%)	0.758	0.384
**Vasopressor (μg/kg/min.)**				
T0	0.41±0.06	0.39±0.06	0.659	0.513
T1	0.36±0.03	0.35±0.03	1.161	0.252
T2	0.33±0.07	0.27±0.06	3.272	0.002*
T3	0.16±0.03	0.14±0.03	2.240	0.030*
**Length of stay**	12(48%)	9(36%)	0.739	0.390
**Mortality**	2(8%)	2(8%)	0.000	1.000

Group (C): received conventional treatment sepsis and placebo tablet, Group (I): received conventional treatment sepsis and Ivardrabine tablet, HD: hemodialysis, eGFR C :estimated glomerular filtration rate based on cystatin C, T0: baseline value, T1: day 1 after beginning of the study. T2: day 1 after beginning of the study T3: day 1 after beginning of the study

## Discussion

AKI is seen in approximately 35% of intensive care patients. The most important causes in more than 50% of AKI cases are sepsis and septic shock. The mortality rate of sepsis-associated AKI varies between 20.9 and 56.13%, depending on the intensity of injury (Palmar et al. 2009; Bagshaw et al. 2007). Early diagnosis and good knowledge of the pathogenesis of AKI, which has become such a big problem in intensive care units, are important.

In this study, we try to assess the therapeutic efficiency of ivardrabine as heart rate reducing agent to improve renal function in septic patient with renal impairment.

Our result shows that group (I) has significant reduction in heart rate starting 24 h after administration of ivabradine therapy (87.04 ± 3.23) and marked reduction (68.13 ± 3.34) on day 3, and this reduction was highly statically significant (*P* < 0.001) in comparison to baseline value (96.84 ± 3.33) in the same group also when compared both groups together (84.64 ± 5.03); this is in agreement with the findings demonstrated by Alexandre Bedet and his colleague (Bedet et al. 2020) on experimental sepsis in mice studying hemodynamic effects (by invasive (left ventricular catheterization) and non-invasive (transthoracic echocardiography)) of ivabradine as compared with a β-blocker (atenolol) during murine peritonitis; they found that heart rate control could be similarly achieved by ivabradine or atenolol, with preservation of blood pressure, cardiac output, and left ventricular systolic function.

In controversy to the prospective, controlled, randomized study carried by Sebastian Nuding et al. (Nuding et al. 2018) on 70 patients with multisystem organ failures to assess the effect of ivardrabine on hemodynamics, disease severity, vasopressor use, they found no significant differences in the primary outcome (the percentage of patients with a heart rate reduction of at least 10 beats/min after 96 h.) between the ivabradine and control groups (*P* = 0.147). After 96 h, the daily median heart rate was reduced by 7 beats/min in the control group and by 16 beats/min in the ivabradine group (*P* = 0.014). No differences in secondary outcomes were observed.

Treating tachycardia in septic shock is controversial. Tachycardia increases cardiac workload and myocardial oxygen consumption. In addition, shortening of diastolic relaxation time and impairment of diastolic function further affect coronary perfusion, contributing to a lower ischemic threshold. Andrea Morelli and his colleague (Morelli et al. 2013) demonstrate the beneficial effect of using esmolol in septic patient with persistent tachycardia to decrease mortality rate, and improve organ function in randomized clinical trail on 77 patients.

As regards renal impairment, there was significant improvement in renal function in group (I) started from day 2 and continue in terms of daily urine output (mean 2141.96 ± 210.83) in day 3 in comparison to baseline value (829.60 ± 105.17) and when compared to group (C) (mean 1838.44 ± 131.88) also in terms of reduction in serum cystatin c level (0.98 ± 0.12 vs. 1.21 ± 0.15) and increase in (eGFR) based on Cystatin C (103.32 ± 6.96 vs. 96.52 ± 6.36) and this reduction was highly statically significant (*P* < 0.001) but unfortunately this is associated by non-significant reduction in the number of patient that need hemodialysis; 2 patients in group I represent 13% while 6 patients in group C represent 16%.

This agrees with a prospective, randomised, controlled study carried out by Andrea Morelli and his colleague (Morelli et al. 2013) which enrolled 77 patients with sepsis with persistent tachycardia to assess the beneficial role of reducing heart rate by esmolol they found that (eGFR) was better maintained in the esmolol group: median AUC of 14 mL/min/1.73 m^2^ (IQR, 6 to 37) than in the control group vs. 2 mL/min/1.73 m^2^ (IQR, − 7 to 20; *P* < .001). The trend remained when excluding patients receiving renal replacement therapy with a median AUC in the esmolol group of 10 mL/min/1.73 m^2^ (IQR, 1 to 35) vs. − 2 mL/min/1.73 m^2^ (IQR, − 9 to 6) in the control group (*P* < .001). During ICU stay, the percentage of patients requiring renal replacement therapy did not differ between groups: 40.3% in the esmolol group vs. 41.6% in the control group.

Vincenzo De Santis et al.’s evaluation (De Santis et al. 2014) demonstrated that ivabradine was able to reduce heart in patients who developed sepsis-related MODS after cardiac surgery, which showed a concomitant increase in end-diastolic volume index (EDVI), stroke volume index (SVI), MAP, and mixed venous saturation (SvO_2_). The hemodynamic improvement resulted in a consistent serum lactate level reduction and norepinephrine dosage.

Also, Miranda and his colleague (Miranda et al. 2017) demonstrate the beneficial role of using ivardrabine in Experimental Sepsis in Twenty-eight golden Syrian hamsters; they found that ivabradine had greater functional capillary density (90 ± 6% of baseline values vs. 71 ± 16%; *P* < 0.001), erythrocyte velocity in capillaries (87 ± 11% of baseline values vs. 62 ± 14%; *P* < 0.001), and arteriolar diameter (99 ± 6% of baseline values vs. 91 ± 7%; *P* = 0.041) at the end of the experiment. Besides that, ivabradine-treated animals had less renal, hepatic, and neurologic dysfunction.

Considering secondary outcomes, there were no statistically significant differences between study groups in terms of length of ICU stay and mortality rate (*P* = 0.390), (*P* = 1.000) respectively. In contrast to the result found in the study by Andrea Morelli and his colleague (Morelli et al. 2013) that reduction of heart rate by esmolol in septic patients had a 28-day mortality rate of 49.6% vs. 80.5% in the control group (*P* < .001). Overall survival was higher in the esmolol group. Multivariable Cox regression analysis revealed the esmolol group allocation (hazard ratio [HR], 0.392; 95% CI, 0.261–0.590; *P* < .001).

## Conclusion

We conclude that reducing the heart rate, using Ivabradine, in patients suffering from sepsis and secondary renal impairment can improve the renal function. Whether this have an impact on improving mortality, more research is needed to investigate this hypothesis.

## Limitations

Frist, we did not use objective method to assess cardiac output or renal blood flow either invasive or non-invasive (as renal blood duplex) because there was a limitation in using resources in our hospital during pandemic of COIVD-19. Secondly, we did not assess the serum level of ivardrabine to assess the pharmacokinetic in such patient which may affect the results.

## Supplementary Information

**Additional file 1.**

## Data Availability

Uploaded master sheet of raw data in additional file under title: title of data.

## Abbreviations

ASICU: Ain Shams Intensive Care Units
AKI: Acute kidney injury
MODS: Multiple organ dysfunction syndrome
eGFR: Estimated glomerular filtration rate
ICU: Intensive care unit
SvO2: Mixed venous oxygen saturation
MAP: Mean arterial pressure
BUN: Blood urea nitrogen
AUC: Area under the curve
EDVI: End-diastolic volume index
SVI: Stroke volume index
